# Boosting CMOS Image Sensors: Exploring Silicon Hydride and Hydrocarbon Molecular Ion Implanted in Epitaxial Wafers

**DOI:** 10.3390/s26206659

**Published:** 2026-10-21

**Authors:** Ayumi Onaka-Masada, Ryo Hirose, Ryosuke Okuyama, Koji Kobayashi, Akihiro Suzuki, Sho Nagatomo, Kazunari Kurita, Takeshi Kadono

**Affiliations:** 1 SUMCO Corporation, 1-52 Kubara, Yamashiro-cho, Imari-shi 849-4256, Japan; aonaka@sumcosi.com (A.O.-M.); rhirose@sumcosi.com (R.H.); rokuyama@sumcosi.com (R.O.); kkobayas@sumcosi.com (K.K.); asuzuki1@sumcosi.com (A.S.); sho_nagatomo@sumcosi.com (S.N.); kurita.kazunari.386@m.kyushu-u.ac.jp (K.K.); 2 Center of Plasma Nano-Interface Engineering, SUMCO Joint Research Department of 3D-Si Integrated Device, Kyushu University, 744 Motooka, Nishi-ku, Fukuoka 819-0395, Japan

**Keywords:** CMOS image sensor, molecular ion, white spot defects, gettering

## Abstract

This paper presents the effect of SiH_x_ and C_2_H_y_ mixed molecular ion implantation into an epitaxial layer with low oxygen concentration on the dark current and white spot defects in complementary metal-oxide-semiconductor (CMOS) image sensor pixels. By utilizing a structure where these mixed molecular ions are implanted into the epitaxial layer, we confirmed a 32% reduction in the number of white spot defects and a 13% reduction in dark current compared to epitaxial wafers without implantation in this initial evaluation. These improvements are attributed to three key mechanisms: (1) enhanced gettering capability for metal impurities, (2) effective suppression of oxygen diffusion into the device’s active layer, and (3) hydrogen diffusion contributing to the passivation of SiO_2_/Si interface defects. Notably, the black dot-like defects and End of Range (EOR) defects formed in the epitaxial layer gettered at lower oxygen concentrations compared to those formed on the Czochralski (CZ) silicon substrate and exhibited higher gettering capacity for Ni. Furthermore, the amount of hydrogen diffusing from EOR defects formed in the epitaxial layer was confirmed to increase.

## 1. Introduction

Complementary metal-oxide-semiconductor (CMOS) image sensors are no longer limited to consumer cameras or smartphones; they are widely used in various imaging systems. As demands for high-sensitivity, high-resolution, and rapid image data processing techniques continue to rise, the need for high-performance imaging systems becomes more pronounced. Recently, the development of advanced CMOS image sensors, such as three-dimensional stacked CMOS image sensors (3D-stacked CIS), has garnered significant attention to enhance the performance of these imaging systems [1,2,3]. However, the use of heavy metal materials in such stacked CMOS image sensors, along with the increased area of interfacial regions due to pixel separation and layered structures, has led to serious technical challenges in reducing dark current [3,4,5].

Dark current is a critical parameter that determines the performance of CMOS image sensors. The primary causes of dark current are believed to include heavy metal impurities contaminated into the silicon wafer, point defects formed within the silicon wafer, and energy levels created in the space charge region due to SiO_2_/Si interface defects [6,7]. These energy levels act as carrier generation centers, becoming the main factor behind dark current generation and leading to large pixel defects referred to as white spots [8].

3D-stacked CISs are characterized by a structure in which the pixel section and data processing functions are stacked through 3D integration and implementation technologies. These components are electrically connected via Cu-Cu hybrid bonding and Cu through-silicon vias (Cu-TSVs) [9,10]. As a result, heavy metal contamination caused by Cu wiring has become a primary contributor to dark current generation [11]. Additionally, energy levels formed in the space charge region due to oxygen-related defects are also considered a factor in dark current generation. This effect is particularly pronounced after ion implantation, as voids (V) and oxygen-related defects formed by ion implantation have been reported to play a significant role [12].

In Czochralski (CZ) silicon substrates, oxygen is dissolved at a concentration of approximately 10^18^ cm^−3^, which out-diffuses into the device active layers during heat treatment in the manufacturing process. Ion implantation is an essential process in silicon device fabrication. Therefore, reducing the out-diffusion of oxygen into device active layers is critical for minimizing dark current. Moreover, as previously mentioned, three-dimensional stacked CMOS image sensors experience an increase in interfacial areas due to pixel separation regions and layered structures. In device isolation regions such as Deep Trench Isolation (DTI), SiO_2_/Si interface defects are formed [13,14]. The primary origin of these interface defects is the dangling bonds of silicon atoms (P_b_ centers) [15,16]. P_b_ centers create energy levels within the space charge region, which means that 3D-stacked CISs—with their increased interfacial areas—are significantly affected by interface-level defects.

To address the above challenges, the authors’ group has developed hydrocarbon molecular ion-implanted epitaxial wafers [17,18]. These wafers exhibit high gettering capabilities against heavy metal impurities introduced during the CMOS image sensor manufacturing process and oxygen outwardly diffused from the silicon substrate during production [19]. Furthermore, it has been reported that hydrogen adsorption–desorption phenomena captured in the hydrocarbon molecular ion implantation regions contribute to the reduction in SiO_2_/Si interface defects [20].

In addition, hydrocarbon molecular ion-implanted epitaxial wafers have been found to significantly reduce white spot defects in CMOS image sensors compared to the conventional gettering method known as Internal Gettering (IG), which uses oxygen precipitates as gettering sinks. The gettering sinks of these hydrocarbon molecular ion-implanted epitaxial wafers are presumed to be 5 nm sized implantation defects (CI complexes) formed by carbon (C) and interstitial silicon (I) [21,22], and the density of these defects depends on the dose of hydrocarbon molecular ions implanted [18]. Thus, it can be said that the gettering capability depends on the amount of carbon implanted. On the other hand, experiments using DLTS (Deep Level Transient Spectroscopy) have revealed that carbon in the silicon wafer forms energy levels within the space charge layer, and carbon diffused into the epitaxial layer, which is the device’s active layer, is a factor that deteriorates the device’s electrical characteristics [23,24,25].

To maintain the high gettering capability of CI complex defects against heavy metal impurities while reducing the carbon concentration being implanted, we have been developing silicon–carbon-based (SiH_x_·C_2_H_y_) mixed molecular ion implantation technology since 2020. This technology is characterized by using two types of mixed molecular ion beams, SiH_x_ ions and C_2_H_y_ ions, for ion implantation. Compared to conventional hydrocarbon molecular ion implantation, the addition of silicon-based molecular ion implantation damage allows for the formation of gettering sinks even when the carbon dose is reduced.

In the SiH_x_·C_2_H_y_ mixed molecular ion-implanted regions, in addition to CI complexes, End of Range (EOR) defects are formed, which have been reported to exhibit high gettering capabilities for Ni impurities [26]. Furthermore, compared to conventional hydrocarbon molecular ion-implanted epitaxial wafers, these regions demonstrate a greater reduction in SiO_2_/Si interface defects caused by hydrogen.

However, the effects of SiH_x_·C_2_H_y_ mixed molecular ion-implanted epitaxial wafers on the product characteristics of CMOS image sensors remain unclear. Particularly, the previously reported gettering capability for Ni and Cu has been based on evaluations under deliberate contamination conditions, making the gettering capability against contamination introduced during the device manufacturing process uncertain.

In this paper, the product characteristics of SiH_x_·C_2_H_y_ mixed molecular ion-implanted epitaxial wafers were evaluated using CMOS image sensors fabricated through the CMOS device manufacturing process at a CMOS image sensor fabrication plant. The evaluation was conducted based on dark current and the number of white spot defects, as derived from Dark Current Spectroscopy (DCS) [27,28,29].

In our previous report, we demonstrated that C_3_H_6_ molecular ion implantation into Epi-Si, a low-oxygen concentration layer, reduced white spot defects in CMOS image sensors compared to implantation into CZ-Si [30]. Therefore, we evaluated the product characteristics using epitaxial wafers in which SiH_x_·C_2_H_y_ mixed molecular ions were implanted into Epi-Si and CZ-Si. We compared these results to wafers without ion implantation and discussed how implanted defects formed during molecular ion implantation into Epi-Si and CZ-Si affect the product characteristics of CMOS image sensors.

## 2. Materials and Methods

**Figure 1:**
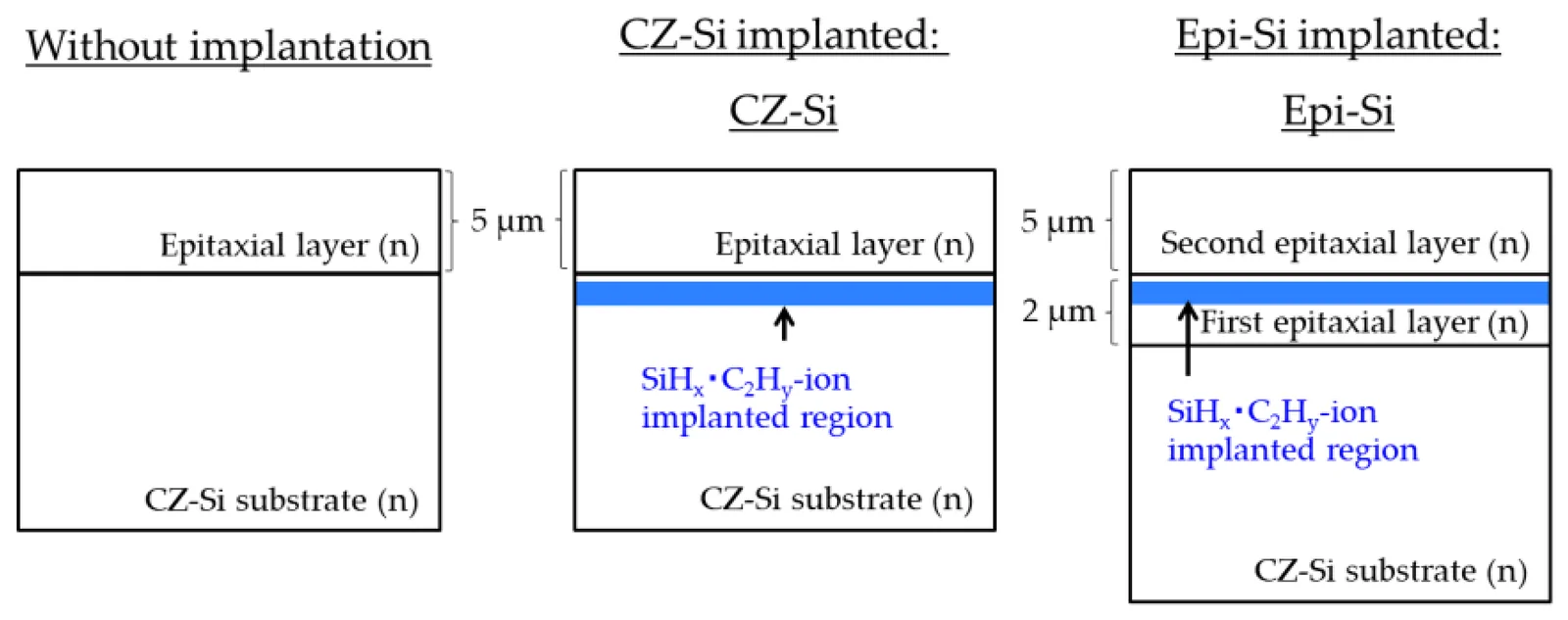
Cross-sectional schematic illustrations of epitaxial wafers used in this study.

Figure 1 shows a cross-sectional schematic diagram of the epitaxial wafers used in this experiment. An n-type CZ-Si wafer doped with phosphorus and an epitaxial Si wafer (Epi-Si) equipped with a 2 μm thick epitaxial layer were utilized. The oxygen and carbon concentrations of the CZ-Si wafer were 1.4 × 10^18^/cm^3^ and 3.0 × 10^16^/cm^3^, respectively. The 2 μm thick epitaxial layer was grown at 1150 °C, and the oxygen and carbon concentrations in the surface layer were less than 1.4 × 10^18^/cm^3^ and 3.0 × 10^16^/cm^3^, respectively. These wafers were subjected to ion implantation of SiH_x_·C_2_H_y_ mixed molecular ions under the following conditions: an acceleration voltage of 80 keV, an ion dose of 5.0 × 10^14^/cm^2^, and a selected mass number (hereafter referred to as Mass value) of 29 during mass separation. The mass 29 consisted of a mixture of SiH_x_- and C_2_H_y_-containing species with mixture rates of 55% and 45%, respectively. This composition corresponds to a silicon dose of 2.75 × 10^14^/cm^2^ and a carbon dose of 4.50 × 10^14^/cm^2^. Subsequently, a 5 μm thick n-type epitaxial layer with a resistivity of 25 Ωcm was grown at 1150 °C.

For the fabricated SiH_x_·C_2_H_y_ mixed molecular ion-implanted epitaxial wafer, a four-transistor pixel-configured CMOS image sensor with a 3 μm pixel size was prototyped on a 65 nm CMOS device production line. During this fabrication, wafers for all experimental conditions were co-processed within a single prototyping lot on the device production line, with one wafer per condition. DCS measurements were conducted on the center die of the prototyped wafers at 60 °C with an integration time of 1 s. Measurements for all conditions were conducted simultaneously under standard operating conditions. In the DCS spectrum obtained from the DCS measurement, the cumulative number of pixels that generated a dark current of 35 ele/sec or more was defined as the number of white spot defects. Additionally, the median of the DCS spectrum was defined as the dark current.

Using the wafer subjected to DCS measurement, the concentration profiles of Ni, carbon, oxygen, and hydrogen in the implanted region were measured by secondary ion mass spectrometry (SIMS) (IMS-7f, CAMECA, Gennevilliers, France). Additionally, analyses focusing on implantation defects were conducted using transmission electron microscopy (TEM) (JEM-F200, JOEL, Tokyo, Japan) and laser-assisted atom probe tomography (L-APT) (LEAP 5000XS, CAMECA, Gennevilliers, France). A specific needle-shaped sample for L-APT, including the target defects, was prepared parallel to the SiH_x_·C_2_H_y_-ion-implanted region using a focused ion beam (FIB) lift-out technique. L-APT measurement was performed on one successfully processed sample using an ultraviolet laser (wavelength: 355 nm). The L-APT data were analyzed using integrated visualization software (IVAS) from CAMECA. Lastly, the number of electrically active defects related to SiH_x_·C_2_H_y_-ion implantation in CZ-Si and Epi-Si was evaluated by room-temperature photoluminescence (RTPL) (MPL300, WaferMasters, San Jose, CA, USA).

## 3. Results and Discussion

### 3.1. Gettering Characteristics of SiH_x_·C_2_H_y_ Mixed Molecular Ion Implanted Epitaxial Si Wafers

#### 3.1.1. Evaluation of Dark Current and White Spot Defects Using the DCS Method

**Figure 2:**
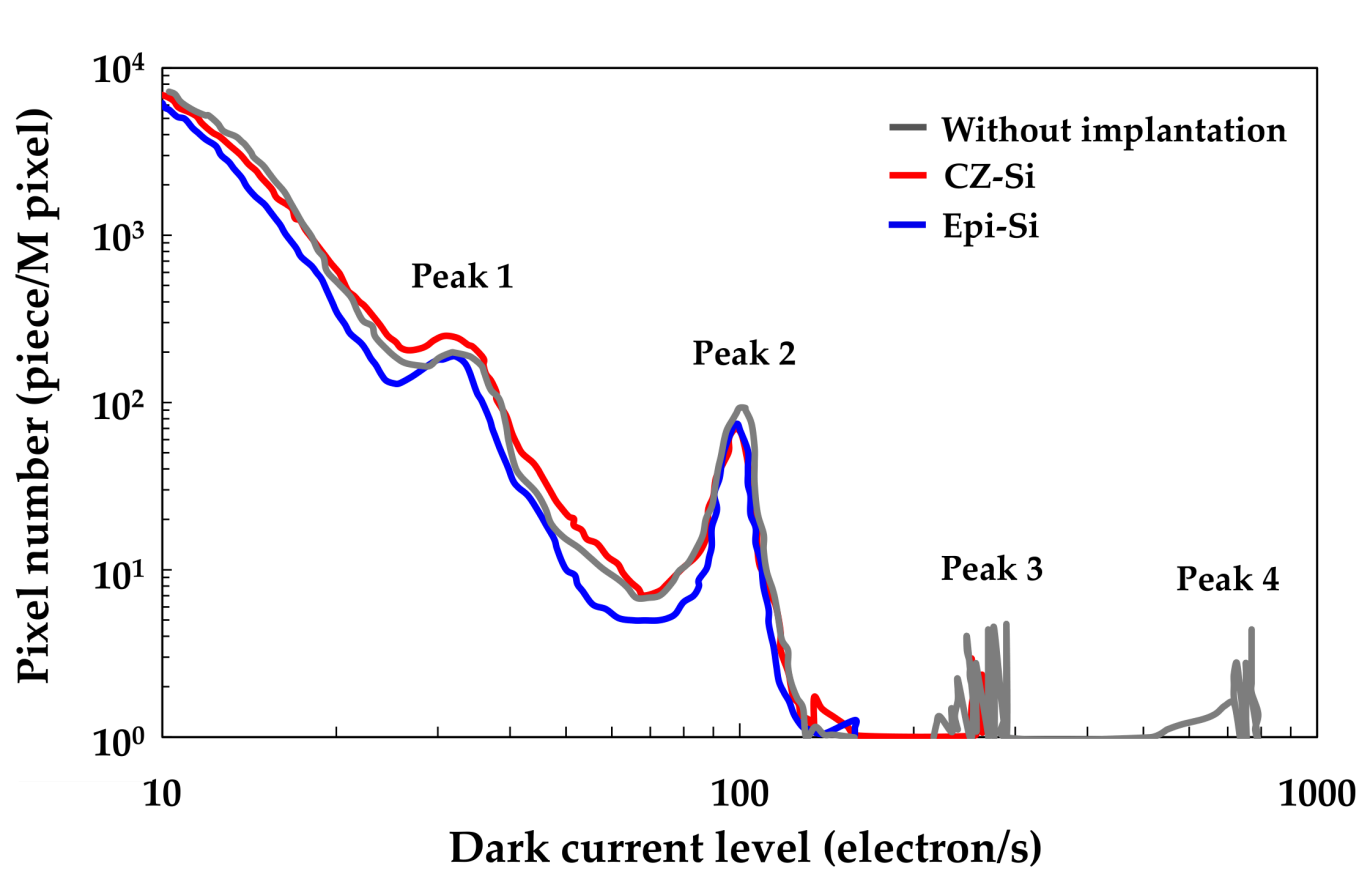
DCS spectra of CMOS image sensors fabricated using epitaxial wafers without ion implantation, and epitaxial wafers with SiH_x_·C_2_H_y_ mixed molecular ions implanted into CZ-Si and Epi-Si.

Figure 2 shows the DCS spectra of epitaxial wafers without implantation and epitaxial wafers implanted with SiH_x_·C_2_H_y_ mixed molecular ions into CZ-Si and Epi-Si. It is evident that the number of white spot defects in the Epi-Si implanted samples is low across all dark current levels. Furthermore, four peaks (Peak 1, Peak 2, Peak 3, and Peak 4) are observed in the DCS spectra, and a reduction in Peak 4 is confirmed for both types of epitaxial wafers implanted with SiH_x_·C_2_H_y_ mixed molecular ions.

The reduction in white spot defects signifies a decrease in the concentration of impurities localized in the active regions of devices. Additionally, the peaks observed at high dark current levels in the DCS spectra are caused by heavy metal impurities [31]. Therefore, the reduction in Peak 4 in epitaxial wafers implanted with SiH_x_·C_2_H_y_ mixed molecular ions is thought to be due to the gettering of heavy metal impurities by defects formed in the SiH_x_·C_2_H_y_ mixed molecular ion-implanted regions, resulting in a decrease in the concentration of heavy metal impurities localized in the active regions of devices.

**Figure 3:**
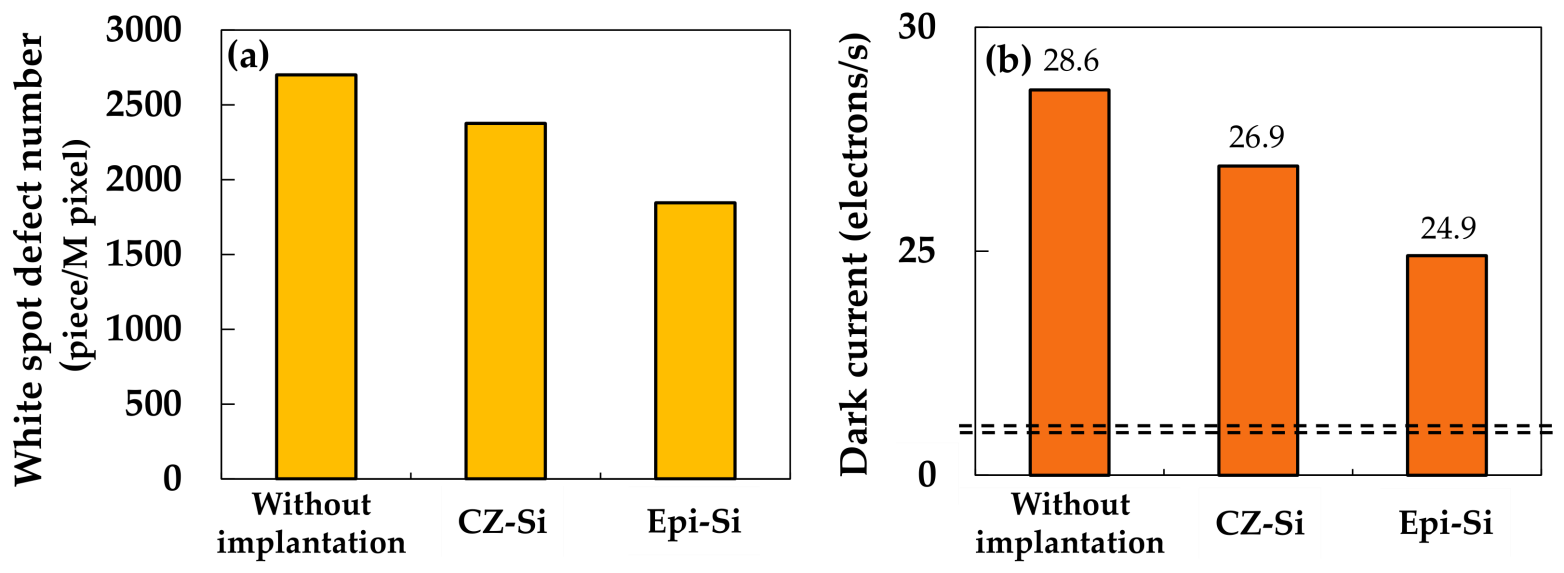
Histograms of (**a**) white spot defects and (**b**) dark current in CMOS image sensors fabricated using epitaxial wafers without ion implantation, as well as epitaxial wafers implanted with SiH_x_·C_2_H_y_ mixed molecular ions into CZ-Si and Epi-Si.

To compare the DCS characteristics, histograms of the number of white spot defects and dark current levels for each epitaxial wafer are shown in Figure 3. From Figure 3a, it is evident that the reduction in the number of white spot defects in the epitaxial wafers implanted with SiH_x_·C_2_H_y_ mixed molecular ions into Epi-Si is the lowest when compared to implantation into CZ-Si. A 32% reduction in the number of white spot defects was observed in the Epi-Si implanted samples compared to epitaxial wafers without implantation.

From Figure 3b, it can be observed that the dark current of Epi-Si implanted samples is significantly reduced compared to the implantation into CZ-Si. As shown in Figure 2, a large number of pixels in the DCS spectra are included in regions with low dark current levels. Since the dark current represents the median value of the DCS spectra, the region controlling the dark current value in the DCS spectra is considered to be the one with low dark current levels. Leakage current occurring in regions with low dark current levels is primarily attributed to defects at the SiO_2_/Si interface [32]. Therefore, the reduction in dark current for implantation into Epi-Si is likely due to the reduction in defects at the SiO_2_/Si interface.

#### 3.1.2. Evaluation of Gettering Ability by SIMS

**Figure 4:**
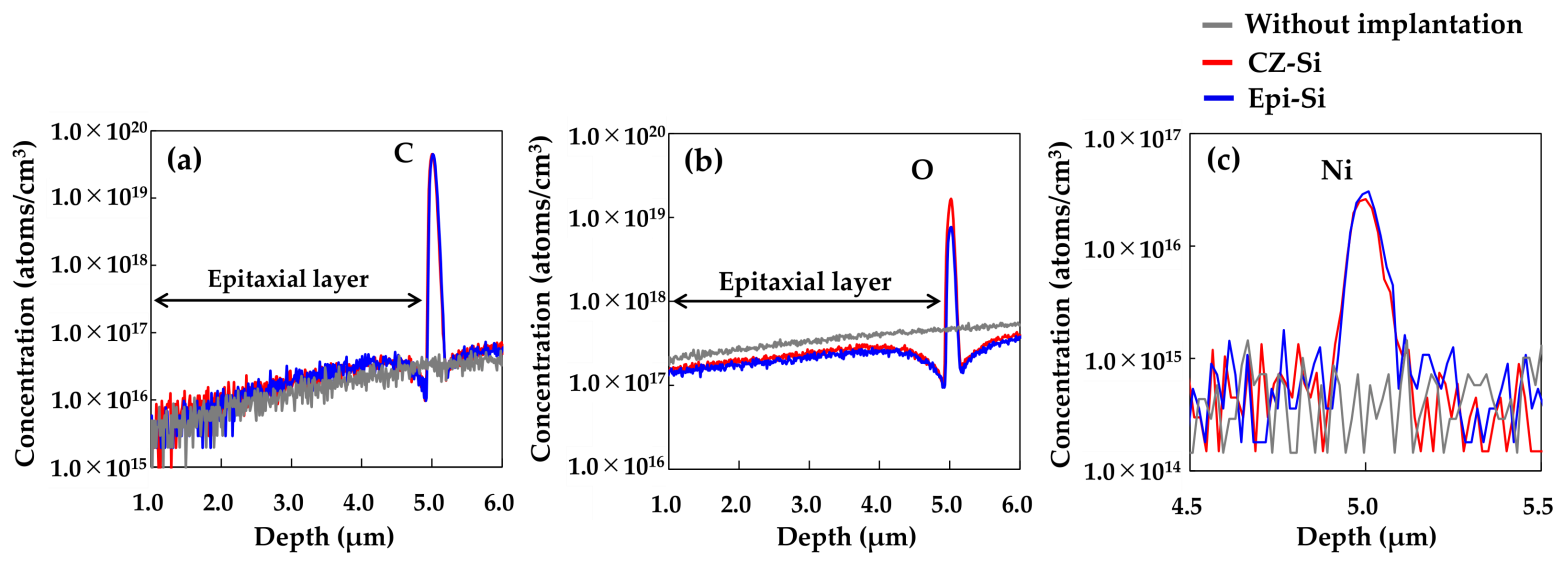
Depth profiles of carbon, oxygen, and nickel concentrations in CMOS image sensors fabricated using various epitaxial wafers: (**a**) carbon, (**b**) oxygen and (**c**) Ni.

Figure 4 shows the depth profiles of carbon (C), oxygen (O), and nickel (Ni) concentrations by SIMS in CMOS image sensors fabricated using epitaxial wafers without ion implantation, as well as epitaxial wafers implanted with SiH_x_·C_2_H_y_ mixed molecular ions into CZ-Si and Epi-Si. Only in epitaxial wafers implanted with SiH_x_·C_2_H_y_ mixed molecular ions into CZ-Si and Epi-Si, the oxygen and nickel are confirmed to be gettered in the molecular ion implantation region formed directly below the epitaxial layer.

In the Ni behavior, no clear Ni signal was detected in the sample without ion implantation, considering the SIMS detection limit. Since all samples were fabricated from the same lot, the same concentration of Ni was introduced during the device process across all samples. Therefore, the non-gettered Ni is considered to have dissolved into the device region, thereby contributing to the generation of white spot defects. Russo et al. [33] conducted DCS evaluations using samples intentionally contaminated with various transition metals. Among their findings, they confirmed an increase in the high dark current tail region within the dark current distribution for Ni-contaminated samples. Furthermore, they noted that such Ni-related dark current degradation can be suppressed by using gettering techniques. Therefore, the peak reduction in the high-dark-current level of the DCS spectrum caused by Ni gettering in this study shows a similar behavior.

Focusing on the CZ-Si and Epi-Si, the concentration of Ni gettered in the molecular ion implantation region is 2.58 × 10^11^ atoms/cm^2^ for CZ-Si implanted samples and 2.95 × 10^11^ atoms/cm^2^ for Epi-Si implanted samples. Despite the carbon concentration in the implantation region being the same, as shown in Figure 4a, the Epi-Si implanted samples exhibit higher Ni gettering capability. Additionally, the oxygen concentration gettered in the implantation region is lower in Epi-Si implanted samples compared to CZ-Si. In other words, lower oxygen concentration in the implantation region corresponds to higher Ni gettering capability in the region. This result aligns well with the difference in gettering capability for heavy metal impurities observed when hydrocarbon molecular ions are implanted into CZ-Si and Epi-Si [30].

**Figure 5:**
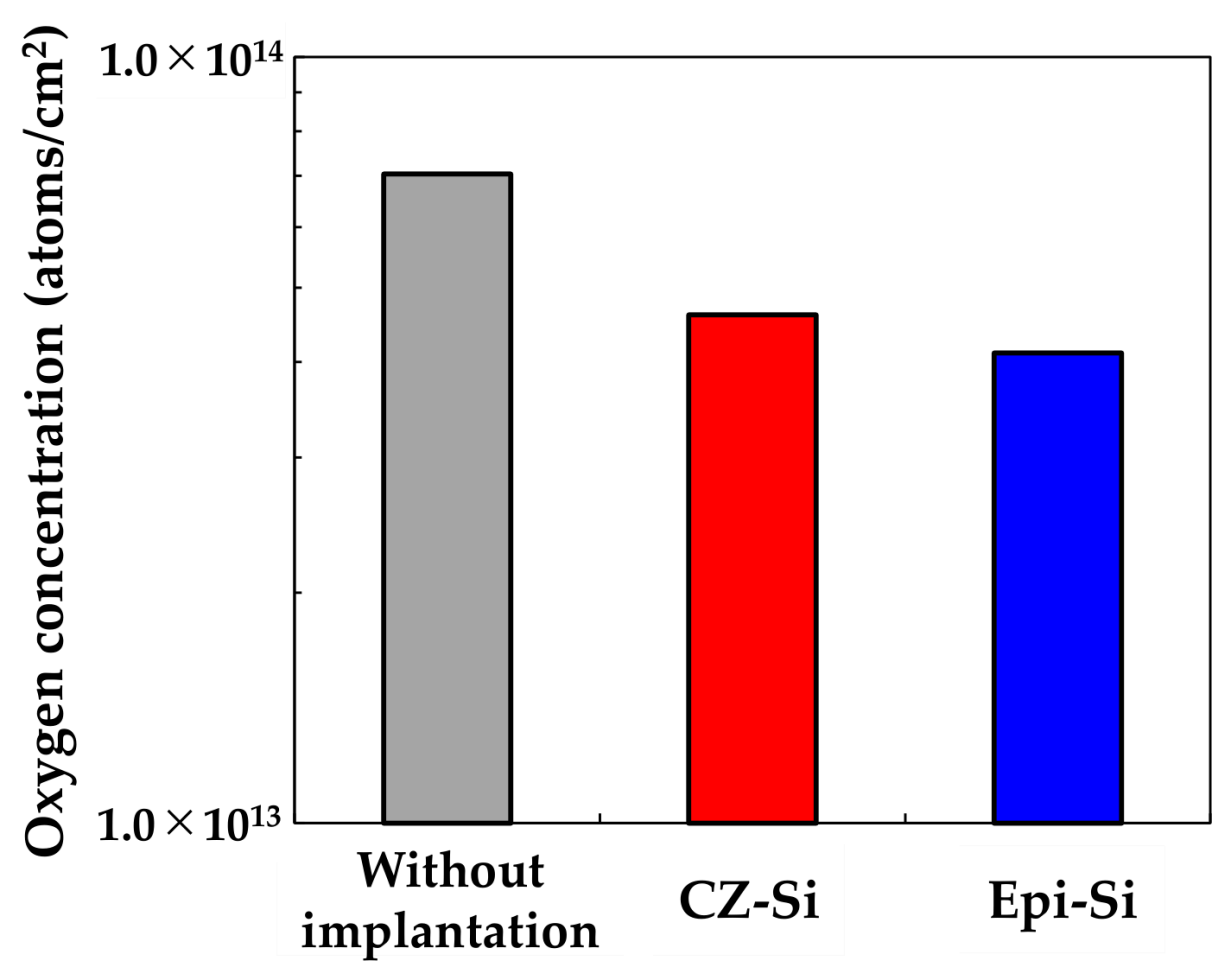
Comparison of oxygen concentration diffused into the device active regions of CMOS image sensor without ion implantation, and CMOS image sensors with SiH_x_·C_2_H_y_ mixed molecular ions implanted into CZ-Si and Epi-Si.

Furthermore, the oxygen concentration diffused into the device active layer during the device process is lower in Epi-Si implanted samples compared to CZ-Si, as shown in Figure 5. This implies that the number of oxygen-induced defects formed in the device active layer is lower in Epi-Si implanted samples than in CZ-Si. Russo et al. [7] reported, based on DCS measurements of CMOS image sensors, that Ni mixed into wafers affects the generation of dark current. Additionally, it has been reported [12] that oxygen diffused into the device active layer forms complex defects with boron (B) and vacancies (V) generated by ion implantation, contributing to the generation of dark current in CMOS image sensors. Therefore, not only the high Ni gettering capability of Epi-Si implanted samples but also the high effectiveness of suppressing oxygen diffusion contribute to the suppression of impurity-related energy level formation in the device active region. As a result, the number of white spot defects is inferred to be reduced.

**Figure 6:**
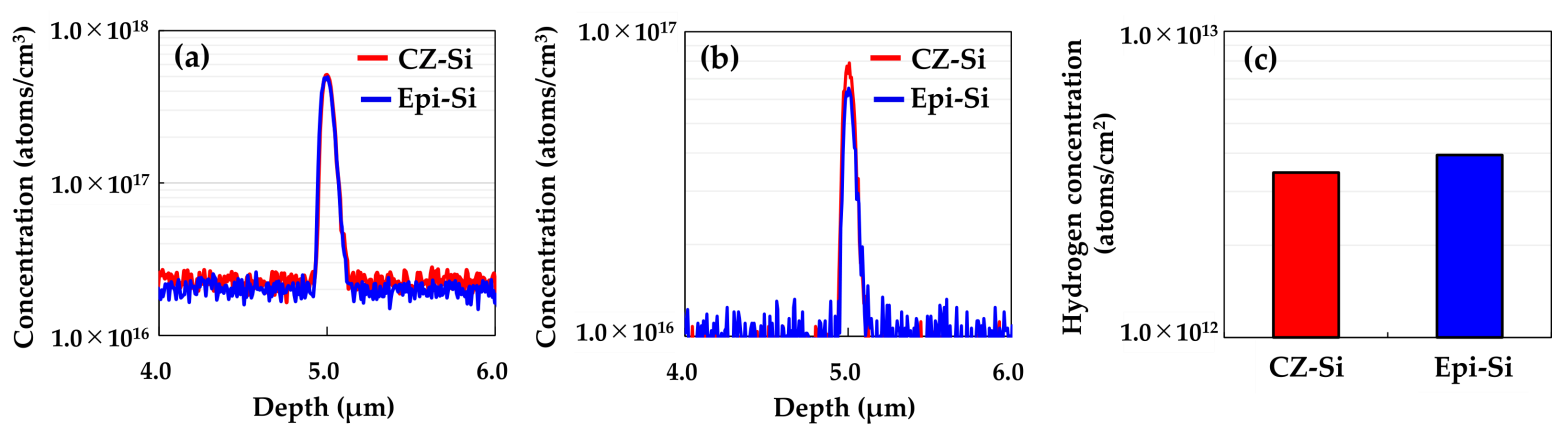
CMOS image sensors fabricated using various epitaxial wafers: (**a**) SIMS depth concentration profiles of hydrogen before device fabrication; (**b**) SIMS depth concentration profiles of hydrogen after device fabrication; (**c**) the amount of hydrogen diffused during the device process.

Figure 6a,b show the depth profiles of hydrogen concentration before and after device fabrication in epitaxial wafers with SiH_x_·C_2_H_y_ mixed molecular ions implanted into CZ-Si and Epi-Si. Based on SIMS analysis, it is observed that there is no significant difference in hydrogen concentration prior to device fabrication. However, after the device fabrication process, the amount of hydrogen trapped in the SiH_x_·C_2_H_y_ mixed molecular ion-implanted region is reduced in the Epi-Si wafers. Figure 6c illustrates the difference in hydrogen concentration before and after device fabrication, representing the amount of hydrogen diffused during the device fabrication process. The amount of hydrogen was calculated by integrating the difference in concentration before and after device fabrication within a depth range of ±0.12 μm from each peak concentration. In this analysis, the background detection limits for hydrogen before and after the device process were 2.0 × 10^16^ and 9.0 × 10^15^ atoms/cm^3^, respectively. This indicates that, compared to CZ-Si wafers, Epi-Si wafers exhibit a greater degree of hydrogen diffusion during the device fabrication process.

Okuyama et al. [20] reported that hydrogen desorbed and diffused from hydrocarbon molecular ion implantation regions could passivate SiO_2_/Si interface defects, thereby reducing defect density. They further demonstrated, using evaluation techniques such as CV and ESR, that hydrogen diffused from the region implanted with SiH_x_·C_2_H_y_ mixed molecular ions plays a role in reducing D_it_ and Pb_0_ center density at the SiO_2_/Si interface [34]. Moreover, Yamaguchi et al. [35] demonstrated that passivation of SiO_2_/Si interface defects by hydrogen diffused from hydrocarbon molecular ion implantation regions could reduce dark current in CMOS image sensors. Therefore, the observed reduction in dark current in Epi-Si implanted samples is thought to be due to the increased amount of hydrogen diffusion during the device process compared to CZ-Si implanted samples.

The adsorption and desorption behavior of hydrogen observed in the hydrocarbon molecular ion implantation region is thought to occur via CI complexes formed by carbon and interstitial Si (I) [18]. On the other hand, in previous studies where hydrocarbon molecular ions were implanted into CZ-Si and Epi-Si, no differences in the amount of hydrogen diffused from the implantation region were observed, which is inconsistent with the current results [30]. From this, it is inferred that the differences in hydrogen diffusion amount observed in the CZ-Si and Epi-Si implanted samples this time are highly likely to occur through defects other than CI complexes.

### 3.2. Evaluation and Analysis of Implantation-Related Defects with SiH_x_·C_2_H_y_ Mixed Molecular Ions

**Figure 7:**
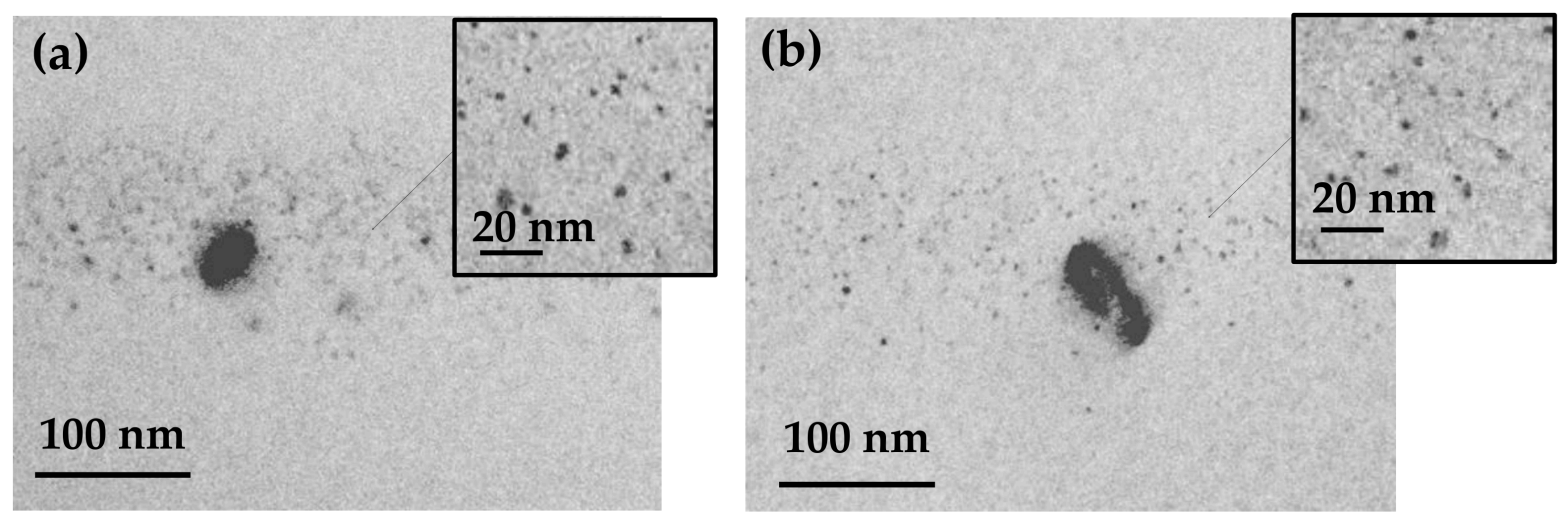
Cross-sectional TEM images of the SiH_x_·C_2_H_y_ mixed molecular ion implantation region for (**a**) CZ-Si and (**b**) Epi-Si.

In Section 3.1, it was demonstrated that the high gettering ability for heavy metals and the high oxygen diffusion suppression effect of epitaxial wafers implanted with SiH_x_·C_2_H_y_ mixed molecular ions into Epi-Si contribute to reducing white spot defects in CMOS image sensors. Furthermore, it was shown that the increase in hydrogen diffusion amount in Epi-Si implanted samples contributes to the reduction in dark current. In order to clarify the nature of defects that contribute to the reduction in white spot defects and dark current in these Epi-Si implanted samples, this section presents the results of analysis on defects formed in the SiH_x_·C_2_H_y_ mixed molecular ion implantation region. Figure 7 shows the cross-sectional TEM images of CMOS image sensors fabricated using epitaxial wafers implanted with SiH_x_·C_2_H_y_ mixed molecular ions into CZ-Si and Epi-Si. In both samples, 5 nm sized black dot-like defects and EOR defects were observed. Previous research [13,14,15,16,28] has revealed that the 5 nm sized black dot-like defects consist of CI complexes and exhibit high gettering ability for heavy metals, oxygen, and hydrogen. The density of black dot-like defects distributed in the implanted region, determined from the field of view of cross-sectional TEM images, was found to be 2.43 × 10^16^/cm^3^ for CZ-Si and 2.42 × 10^16^/cm^3^ for Epi-Si. For these calculations, the sample thickness was 0.5 μm, and the numbers of black dot-like defects within the observation areas of 10,038.8 nm^2^ for the CZ-Si sample and 10,001.3 nm^2^ for the Epi-Si sample were used. This indicates that the difference in defect density does not account for the variation in gettering capability. Furthermore, the observed EOR defects were identified as dislocation loops on the {111} planes, and it was found that the gettering ability for Ni increases with the density of EOR defects [26].

The density of EOR defects in CZ-Si and Epi-Si implanted samples, determined from plan-view TEM observation, was 1.48 × 10^10^/cm^3^ and 1.40 × 10^10^/cm^3^, respectively. For these calculations, the sample thickness was 1.0 μm, and the numbers of EOR defects within the observation areas of 67.6 μm^2^ for the CZ-Si sample and 71.4 μm^2^ for the Epi-Si sample were used. This confirms that the difference in defect density does not contribute to variation in gettering ability for EOR defects. In a previous study involving the implantation of hydrocarbon molecular ions, it was demonstrated using the L-APT method that differences in oxygen concentration in black dot-like defects affect the gettering ability for heavy metal impurities [30]. To clarify the nature of defects contributing to the reduction in white spot defects and dark current in Epi-Si implanted samples, the distribution of oxygen in black dot-like defects and EOR defects was investigated using the L-APT method.

**Figure 8:**
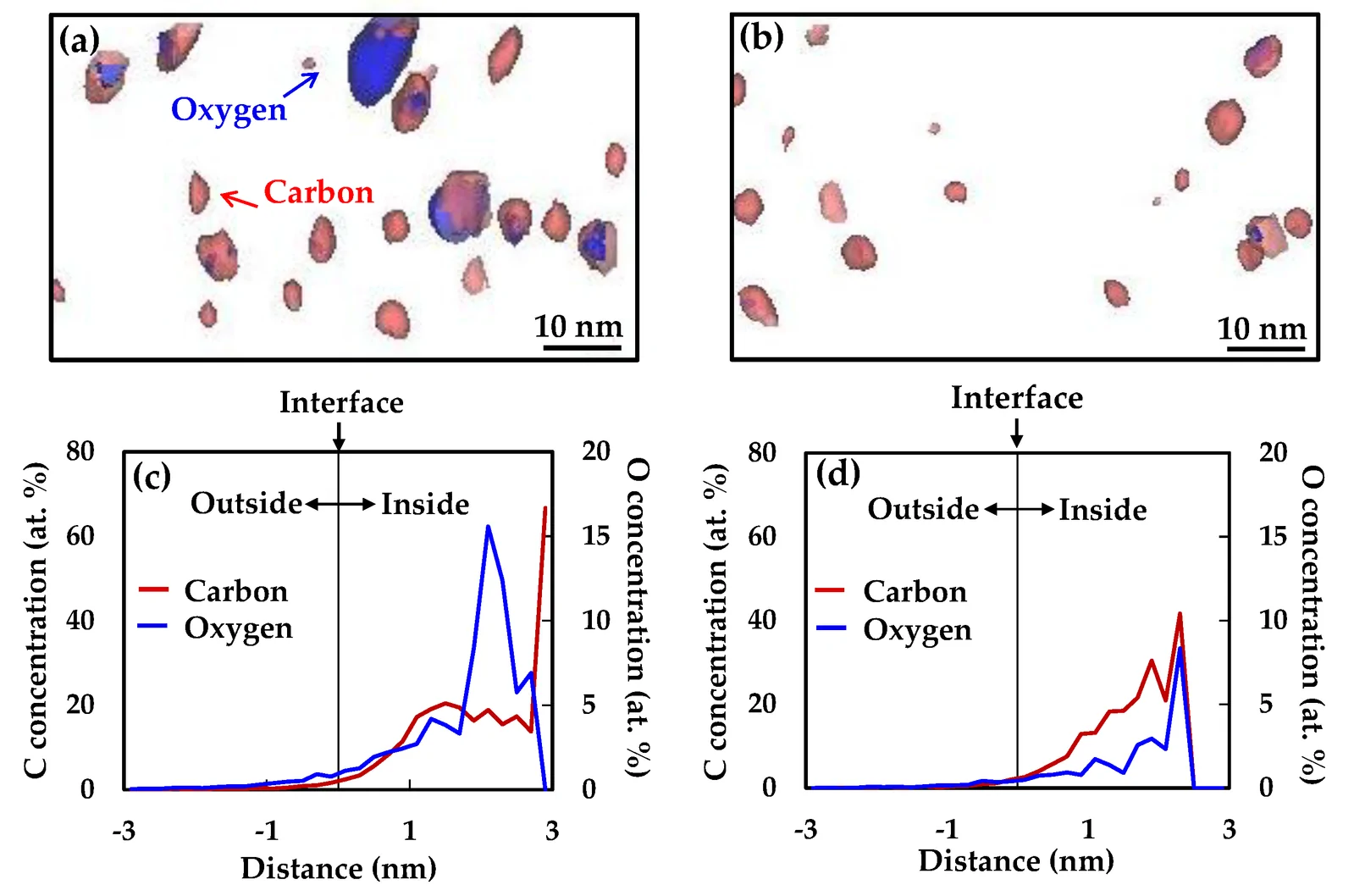
The black dot-like defects observed using L-APT are shown in (**a**) CZ-Si and (**b**) Epi-Si, featuring iso-concentration surfaces of C at 1.8 at% and O at 1.5 at%, as well as (**c**) CZ-Si and (**d**) Epi-Si concentration profiles in the vertical direction near the carbon aggregates. The volume of the extracted map is 75 × 45 × 30 nm^3^.

Figure 8a,b show the isoconcentration surfaces of C 1.8 at% and O 1.5 at% observed in black dot-like defects in CZ-Si and Epi-Si. Isoconcentration surfaces are a representative analytical method in L-APT analysis, and the surfaces of C 1.8 at% and O 1.5 at% depict regions containing 1.8 at% C and 1.5 at% O. This analysis allows for a visual understanding of atomic distribution and their interfaces. From Figure 8, it can be observed that carbon forms aggregates in both CZ-Si and Epi-Si. These carbon aggregates correspond to the black dot-like defects of 5 nm size observed in TEM and are presumed to be CI complexes. Moreover, Figure 8c,d show the concentration profiles of C and O focused on a typical black dot-like defect formed in each implantation sample of CZ-Si and Epi-Si. In the CZ-Si implantation sample, it was found that a large amount of oxygen, with a peak concentration of 15.5 at%, is contained within the defect. On the other hand, in the Epi-Si implantation sample, a smaller amount of oxygen was detected within the defect, with a lower peak concentration of 8.3 at%. This difference aligns with the oxygen concentration in the SiH_x_·C_2_H_y_ implanted region shown in the SIMS profiles in Figure 4b. Therefore, focusing on the oxygen concentration in the defects, it was shown that the morphology of the black dot-like defects formed by SiH_x_·C_2_H_y_ mixed molecular ion implantation differs between CZ-Si and Epi-Si. Defects formed in Epi-Si by hydrocarbon molecular ion implantation are CI complexes that do not contain oxygen. It is known that the oxygen within the defects acts as a factor inhibiting the gettering capability for heavy metal impurities [30]. The morphology of black dot-like defects formed by SiH_x_·C_2_H_y_ mixed molecular ion implantation in CZ-Si and Epi-Si closely resembles the morphology of black dot-like defects formed by hydrocarbon molecular ion implantation. From this, it can be said that the formation of black dot-like defects that do not contain oxygen or have a low oxygen concentration strongly depends on the oxygen concentration of the substrate being implanted, rather than on the type of ions being implanted. It is considered that black dot-like defects with low internal oxygen concentration are a factor in improving the gettering capability for heavy metal impurities.

**Figure 9:**
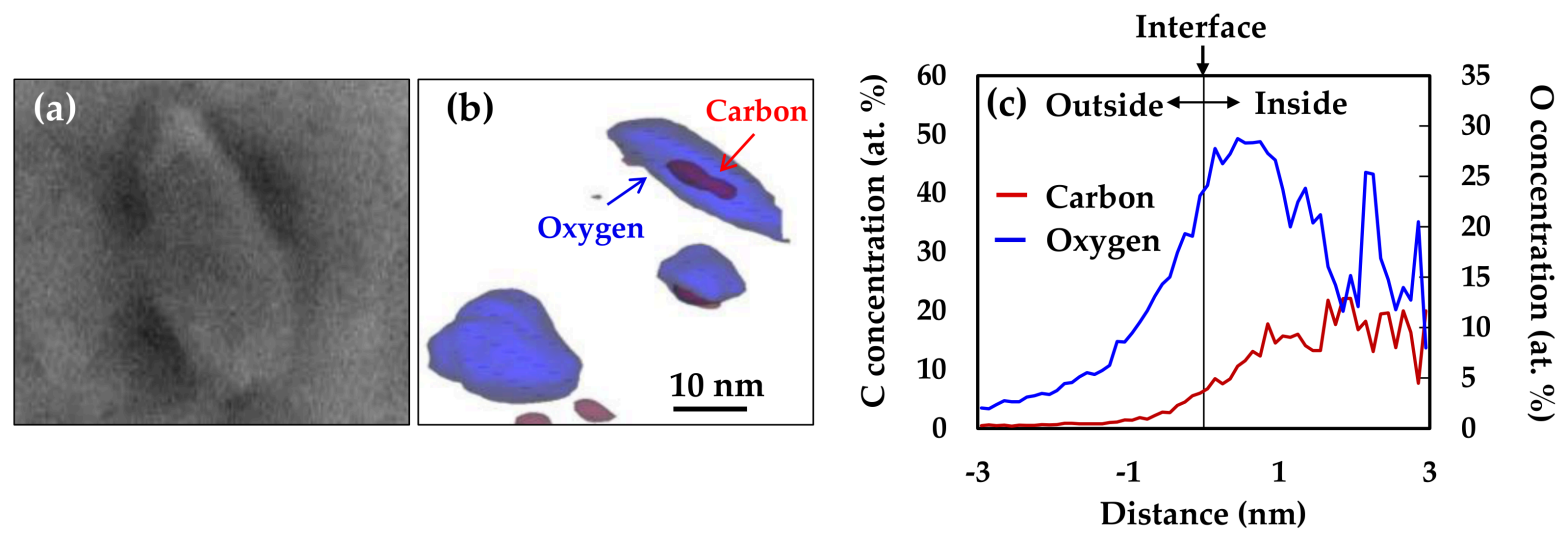
(**a**) STEM images of EOR defects in CZ-Si, (**b**) iso-concentration surface for C at 4.0 at% and O at 3.0 at% around the EOR defects in CZ-Si obtained by L-APT, and (**c**) concentration profiles of C and O in the representative carbon aggregates in EOR defects in CZ-Si. The volume of the extracted map is 50 × 45 × 30 nm^3^.

**Figure 10:**
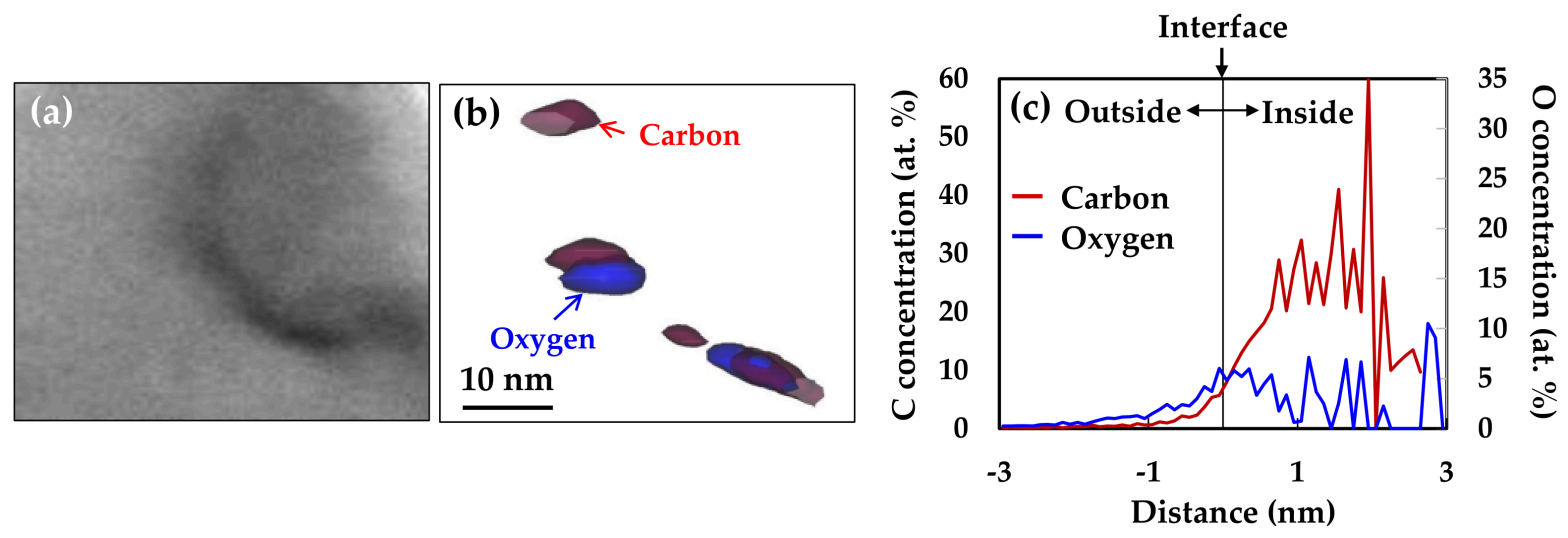
(**a**) STEM images of EOR defects in Epi-Si, (**b**) iso-concentration surface for C at 4.0 at% and O at 3.0 at% around the EOR defects in Epi-Si obtained by L-APT, and (**c**) concentration profiles of C and O in the representative carbon aggregates in EOR defects in Epi-Si. The volume of the extracted map is 50 × 40 × 30 nm^3^.

Next, Figure 9 and Figure 10 show the iso-concentration surfaces of C at 4.0 at% and O at 3.0 at% observed in the EOR defects of CZ-Si and Epi-Si, along with the concentration profiles of the defects. From Figure 9b and Figure 10b, it is evident that high concentrations of carbon segregate at the edges of the EOR defects in both CZ-Si and Epi-Si, forming a ring-like distribution. On the other hand, oxygen does not segregate at the edges of the EOR defects but rather inside or around the area where carbon is highly concentrated. Furthermore, the oxygen concentrations differ between CZ-Si and Epi-Si, with peak concentrations of 28.7 at% and 7.1 at%, respectively, as seen in Figure 9c and Figure 10c. Carbon segregated in extended defects has been reported to alleviate the strain fields present around the defects [36]. However, Figure 9c and Figure 10c indicate that the carbon concentration segregated in the EOR defects of CZ-Si and Epi-Si does not show differences that would impact the metal impurity gettering capability. On the other hand, dangling bonds associated with dislocations and stacking faults also serve as effective gettering sites for metal impurities [37]. Medernach et al. [38] reported that oxygen in defects deactivates the dangling bonds present in defects, thereby reducing the gettering capacity for heavy metal impurities.

Namely, oxygen segregated in EOR defects can also be a factor in hindering the gettering of heavy metal impurities. Therefore, the low concentration of oxygen segregated in EOR defects in Epi-Si implanted samples is considered one of the factors contributing to the improvement of Ni gettering capability in Epi-Si implanted samples.

Furthermore, Okuyama et al. [39] suggested that carbon segregated at EOR defects serves as adsorption and desorption sites for hydrogen. Figure 9c demonstrates that in the EOR defects formed after implanting SiH_x_·C_2_H_y_ mixed molecular ions into CZ-Si, a high concentration of oxygen exists around the carbon segregated at the defect edges. This segregation region of carbon and oxygen overlaps, suggesting the possibility that carbon forms complexes with oxygen or that carbon is gettering oxygen.

Therefore, in CZ-Si implanted samples, it is suggested that defects containing oxygen serve as adsorption and desorption sites for hydrogen. Previous research has reported the thermal stability of oxygen–molecular hydrogen complexes (O–H_2_) [40]. On the other hand, Epi-Si implanted samples showed a lower amount of oxygen segregation in EOR defects compared to CZ-Si implanted samples, as shown in Figure 10c.

Moreover, in Epi-Si implanted samples, the higher presence of individual carbon not associated with oxygen suggests that carbon serves as adsorption and desorption sites for hydrogen. In other words, the adsorption and desorption sites for hydrogen differ between Epi-Si-implanted samples and CZ-Si-implanted samples, and this difference is presumed to contribute to the variation in the amount of hydrogen diffusion observed in CZ-Si- and Epi-Si-implanted samples.

**Figure 11:**
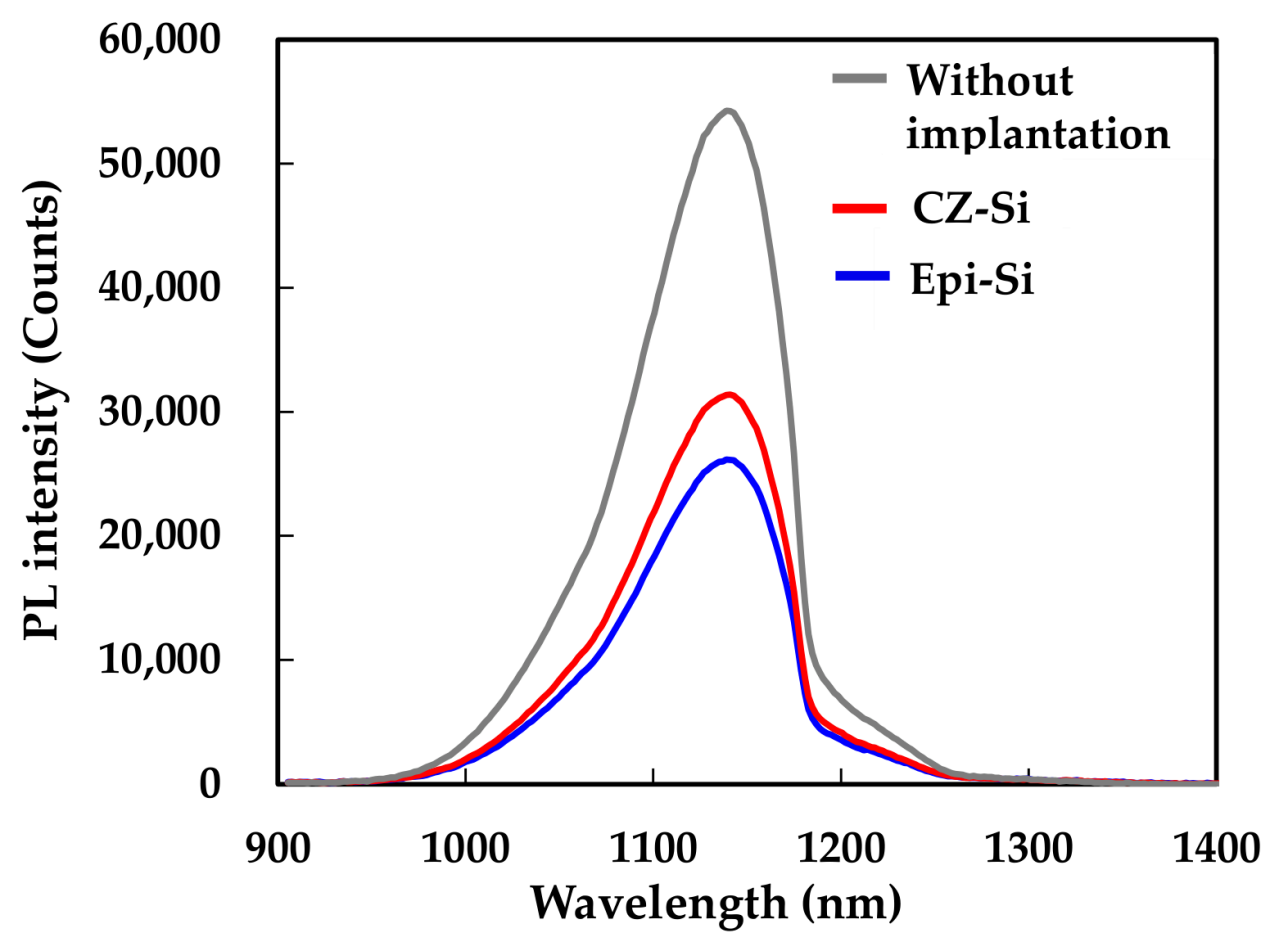
RTPL spectra under 827 nm excitation for epitaxial wafers with SiH_x_·C_2_H_y_ mixed molecular ions implanted into CZ-Si and Epi-Si, as well as for without implanted epitaxial wafers.

Finally, RTPL analysis was conducted on the wafers prior to device formation to investigate the impact of differences in oxygen concentration at defects acting as gettering sinks, revealed through L-APT analysis, on the electrical properties and gettering capability. Figure 11 shows the RTPL analysis results of epitaxial wafers implanted with SiH_x_·C_2_H_y_ mixed molecular ions into CZ-Si and Epi-Si, as well as those of un-implanted epitaxial wafers. The wavelength of the laser used in this measurement was 827 nm, and its penetration depth was 10 μm. As shown in Figure 11, a peak near a wavelength of 1100 nm was observed in all wafers after epitaxial growth.

This peak corresponds to the inter-band transition luminescence of silicon, and the peak luminescence intensity depends on the high quality of crystallinity. It was found that the intensity of inter-band transition luminescence is lowest in the implanted Epi-Si sample. This result indicates that the implanted defects contributing to carrier recombination are more abundant in the implanted Epi-Si sample. Thus, the difference in luminescence intensity between the implanted CZ-Si and Epi-Si samples in the RTPL measurements is considered to reflect the difference in the number of electrically active defects contributing to the gettering of heavy metal impurities, oxygen, and hydrogen. In other words, the number of electrically active defects in the implanted CZ-Si and Epi-Si samples differs even before device formation, which is attributable to the differences in oxygen concentration in CI complexes and EOR defects observed by L-APT. Therefore, despite the comparable densities of black dot-like defects and EOR defects observed in the TEM images after device formation, the Epi-Si sample has a higher gettering capability for heavy metal impurities than the CZ-Si sample.

Based on these results, the reduction in white spot defects and dark current in epitaxial wafers implanted with SiH_x_·C_2_H_y_ mixed molecular ions into Epi-Si can be explained by their high gettering ability for heavy metal impurities, strong diffusion suppression effect for oxygen, and increased diffusion of hydrogen. The gettering effect for heavy metal impurities and oxygen diffusion suppression are dependent on the oxygen concentration present in CI complexes and EOR defects formed in the implantation region. It is concluded that the low oxygen concentration in defects of Epi-Si implanted samples contributed to an increase in the number of defects. Furthermore, in Epi-Si implanted samples, hydrogen desorption from carbon segregated in EOR defects becomes dominant, leading to an increase in the diffusion of hydrogen during the device process.

## 4. Conclusions

A CMOS image sensor, fabricated through the CMOS device manufacturing process at the CMOS image sensor fabrication plant, was utilized to evaluate the sample characteristics of epitaxial wafers. These wafers, implanted with SiH_x_·C_2_H_y_ mixed molecular ions into CZ-Si and Epi-Si, were analyzed using the number of white spot defects and dark current obtained through the DCS method as evaluation indicators.

In this initial evaluation, we confirmed a 32% reduction in white spot defects and a 13% reduction in dark current compared to non-implanted wafers by utilizing a structure where SiH_x_·C_2_H_y_ mixed molecular ions are implanted into the epitaxial layer. The reductions in white spot defects and dark current are attributed to the high gettering ability for heavy metal impurities and high diffusion suppression effect for oxygen of the Epi-Si implanted wafers, as well as an increase in the diffusion amount of hydrogen.

The increase in hydrogen diffusion in Epi-Si implanted wafers is a unique result of the implantation of SiH_x_·C_2_H_y_ mixed molecular ions. Furthermore, L-APT analysis confirmed the formation of CI complexes and EOR defects with low oxygen concentrations inside and at the edges of defects in Epi-Si implanted wafers.

It is considered that the improvement in the gettering ability for heavy metal impurities is contributed by both the CI complexes and EOR defects with low oxygen concentrations, while the increase in hydrogen diffusion is attributed to the presence of EOR defects with low oxygen concentrations.

However, these findings are limited to preliminary results from a single wafer of a single fabrication lot. To confirm the reproducibility and assess statistical variations across different wafers and lots, further evaluations in future mass-production phases will be required.

## Data Availability

The data presented in this study are available on request from the corresponding author. The data are not publicly available because of confidentiality concerns.
